# Methods for integrating high-resolution land, climate, and infrastructure scenarios in a hydrologic simulation model

**DOI:** 10.1016/j.mex.2019.10.010

**Published:** 2019-10-16

**Authors:** Sujithkumar Surendran Nair, Ryan A. McManamay, Christopher R. Derolph, Melissa Allen-Dumas

**Affiliations:** a Environmental Science Division, Oak Ridge National Laboratory, Oak Ridge, TN, 37922, United States; b Urban Dynamics Institute Oak Ridge National Laboratory, Oak Ridge, TN, 37922, United States; c Department of Environmental Science, Baylor University, Waco, TX, United States; d Computational Sciences and Engineering Division, Oak Ridge National Laboratory, Oak Ridge, TN, 37922, United States

**Keywords:** High resolution spatially explicit modeling of land-energy-water nexus in cities: Integrating socioeconomic and climatic drivers, Human infrastructure, Urbanizing river basins, Climate drivers, Regional hydrology, Integrated modeling framework

## Abstract

Global alterations of the hydrologic cycle by humans have led to alarming rates of water shortages and irreversible ecosystem change. Our ability to manage water resources lies in accurately modeling water availability at scales meaningful to management. Although hydrologic models have been used to understand the implications of future climate and land cover change on regional water availability, many modeling approaches fail to integrate human infrastructures (HI) with bio-geophysical drivers to facilitate sustainable regional water resource management. This paper presents an integrated framework, inclusive of modeling and data needs, to quantify the effects of both bio-geophysical and HI influence on regional surface water hydrology. The framework enables the integration of high spatial and temporal anthropogenic alterations of water availability for identifying hot-spots and hot-moments of hydrological stresses within individual river-segments using a hydrologic simulation model, Soil and Water Analysis Tool (SWAT).

• A high-resolution river network for the study region with a greater spatial granularity compared to contemporary SWAT applications attempted to account for HI.
• The anthropogenic influence on water balance for each river segment was estimated using data on human infrastructures, such as water intakes, power production facilities, discharges, dams, and land transformation.

## Graphical abstract

**Figure:**
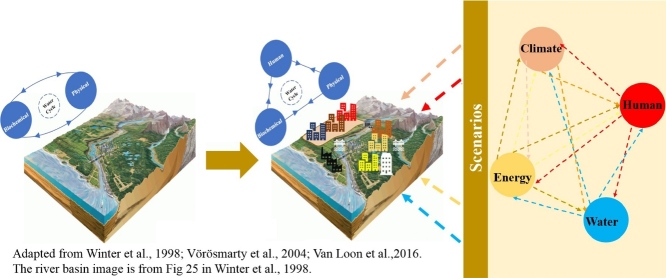

**Specification Table**

**Table:** 

Subject Area:	Environmental Science
More specific subject area:	Urban-Water-Energy Nexus
Method name:	High resolution spatially explicit modeling of land-energy-water nexus in cities: Integrating socioeconomic and climatic drivers
Name and reference of original method:	If applicable, include full bibliographic details of the main reference(s) describing the original method from which the new method was derived. Mehran et al. [54] Compounding impacts of human-induced water stress and climate change on water availability. Sci Rep 7:6282 AghaKouchak et al. [2] Water and climate: recognize anthropogenic drought. Nature 524(7566):409–411 Hanasaki et al. [52] A global water scarcity assessment under shared socio-economic pathways part 1: water use. Hydrol Earth Syst Sci 17(7):2375–2391 Jaramillo and Nazemi [53] Assessing urban water security under changing climate: challenges and ways forward. Ashraf et al. [1] Compounding effects of human activities and climatic changes on surface water availability in Iran Climatic Change 152: 379. Van Loon, A. F., Gleeson, T., Clark, J., Van Dijk, A. I. J. M., Stahl, K., Hannaford, J., Di Baldassarre, G., Teuling, A. J., Tallaksen, L. M., Uijlenhoet, R., Hannah, D. M., Sheffield, J., Svoboda, M., Verbeiren, B., Wagener, T., Rangecroft, S., Wanders, N., and Van Lanen, H. A. J.: Drought in the Anthropocene, Nature Geosci., 9, 89–91, doi:10.1038/ngeo2646, 2016. McManamay, Ryan A., DeRolph, Christopher R., Surendran-Nair, Sujithkumar, and Allen-Dumas, Melissa. Spatially explicit land-energy-water future scenarios for cities: Guiding infrastructure transitions for urban sustainability. United States: N. p., 2019. Web. doi:10.1016/j.rser.2019.06.011.
Resource availability:	NA

## Method details

Human demands on global freshwater resources are increasing at an alarming rate [1,2]. The unprecedented wave of urban-centric population growth could exacerbate the loss of water resources due to extensive infrastructure expansion required to meet the increased demands imposed by cities. Recent studies have cautioned that continued changes to hydrological processes could lead to unprecedented regime shifts, such as anthropogenic drought [[3], [4], [5],^1^]. Further, some regions of the world will experience precipitation shortages, leaving many cities across the globe with a “double-exposure” of water scarcity, both human created and climate change generated. These compounded stresses pose a significant challenge for urban water managers. However, if managers have predicted water shortages using the best available data and tools, then cities can effectively plan, prepare, invest, and adapt in advance to minimize the impacts of water scarcity. Specifically, there is a great need to model the effects of detailed human infrastructures (HI), water infrastructures and energy infrastructures on regional hydrology and potential feedbacks of water shortages on infrastructure resilience. In this context, computer-based hydrologic simulation models that can characterize the spatial distribution of anthropogenic stressors at right resolution and appropriate scale can be useful in representing HI at different scales.

**Fig. 1:**
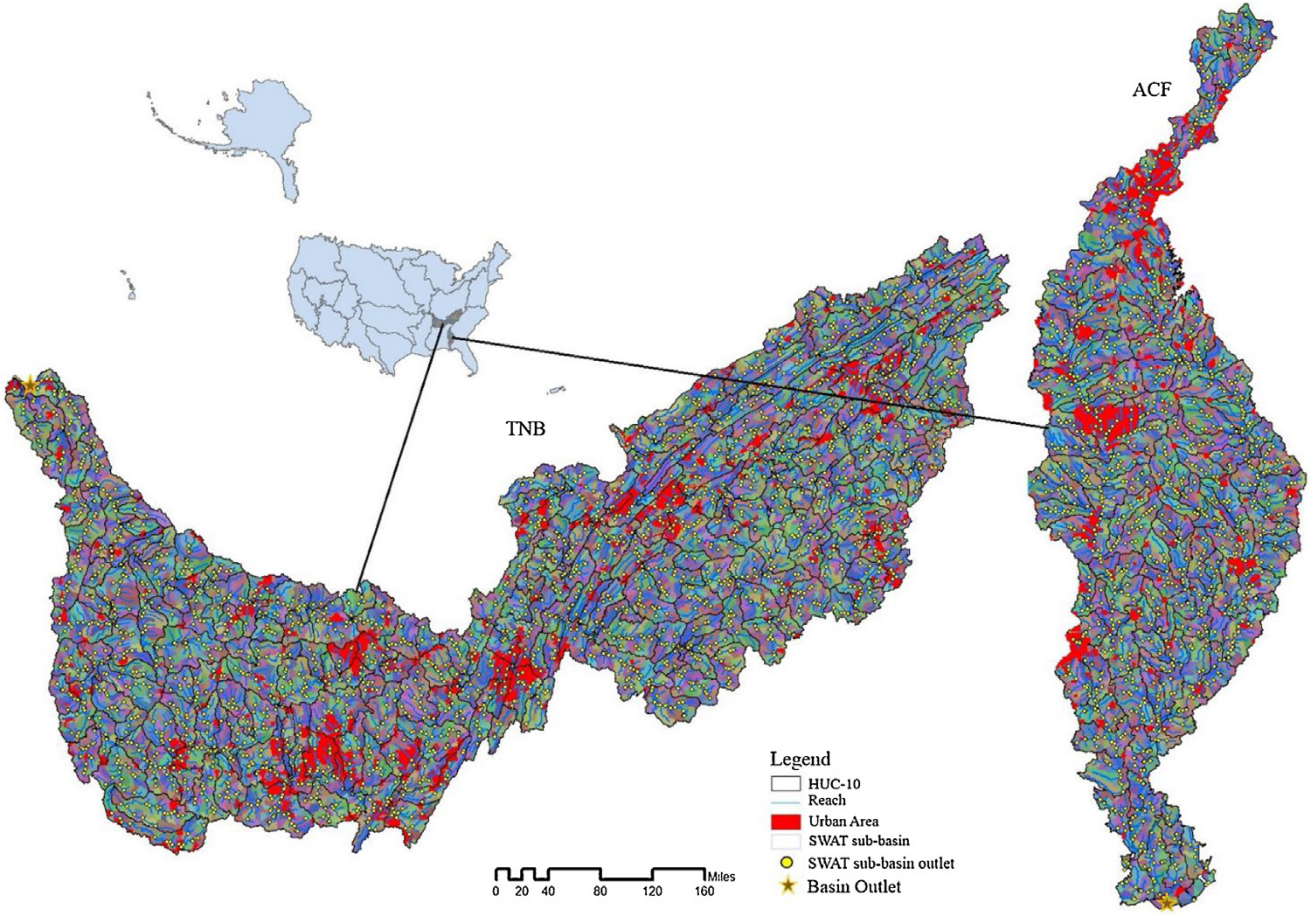
The study areas – 1. Tennessee River basin (TNB) and 2. Apalachicola-Chattahoochee-Flint River (ACF).

Most current hydrologic model applications give little or no attention to explicit inclusion of HIs [1,4,5,52]. The lack of direct representation of human agents in these applications have elicited multiple calls among the socio-hydrologic research community for better-suited hydrologic models capable of examining the compounding effects of HI in water management on the hydrological cycle [3,[6], [7], [8],^56^]. However, many hydrologic models provide sufficient flexibility to accommodate detailed water infrastructures, such as point-source discharge, water abstraction, storage, flow diversion, and return flows. This suggests that much of the hydrologic modeling community may be unaware of the flexibility provisioned by existing platforms, or, in the least, unfamiliar with methods or available data for incorporating HI in a hydrologic framework. Therefore, there is a need for a generalizable hydrologic modeling framework that takes advantage of current hydrologic modeling platforms to accommodate both natural and high-resolution anthropogenic drivers. This paper presents such a framework to quantify the impacts of both natural and HIs on the surface water budget at high spatial resolutions, specifically that of stream segments. To provide an example of the methodology, we apply the framework to two basins with different levels of urban development in the southeastern US: 1) the Tennessee River basin (TNB, 105,870 km^2^) and 2) the Apalachicola-Chattahoochee-Flint River (ACF, 52719.21 km^2^) Basin (Fig. 1). These basins are used to develop the generalized hydrologic modeling framework accommodating natural features and HI, such as urban landscapes (shown by red color in Fig. 1.).

## Framework for incorporating HI in hydrologic modeling platforms

Humans alter water availability in multiple ways related to storage, redistribution, and consumption of water resources [9,10], many of which profoundly modify regional hydrology [[11], [12], [13], [14]]. Alterations to hydrology can be reflected in water budget changes at the scale of local catchments and stream segments, each of which have unique human-natural ecosystem structures with individual responses to shifting water availability. Capturing the spatial fidelity of hydrologic responses at this scale is extremely important for understanding infrastructure resilience to water stress, and potential biological responses. Both the TNB and ACF river basins are heavily impacted by human agents, especially where flow is dammed, diverted and redistributed to meet multitude conflicting societal and ecosystem needs including flood prevention, energy production, consumptive uses, and maintenance of natural ecosystem function. Therefore, accurate representation of the HI is critical for simulating streamflow, especially when the objective of modeling is to understand the impacts of HI, and subsequent vulnerabilities arising from population growth, infrastructure expansion, and changes to climate and land cover. We developed an approach with five sequential stages for effectively integrating HI in an existing hydrologic model.

1 Select a hydrologic simulation model flexible enough to account for HI.
2 Conduct initial set-up and data requirements
3 Identify possible entry points in the selected model to represent HI
4 Determine the granularity of model needs to accommodate HI
5 Generate current and future space-time data (at resolutions meaningful to the model) for each of HI and for each of the identified entry points.
6 Calibrate and validate the model.
7 Iterative calibration and validation based on accommodation of complex HI structure
8 Simulation
9 Implementing future scenarios

### Selecting a hydrologic model and initial set-up

We selected the Soil and Water Assessment Tool (SWAT) for demonstrating the proposed unified hydrologic modeling framework by integrating natural climatological and geophysical processes with HI to accurately represent local and regional hydrology. Other popular hydrology models could also be useful for this purpose and include WASP (Water Quality Analysis Simulation Program; [15]), MIKE 11 [16], HSPF (Hydrological Simulation Program-FORTRAN; [17]). However, we selected SWAT because of its acceptance within the hydrologic community across the globe, specifically in the US, and its applications boasts more than 1500 peer-reviewed publications. Additionally, Kannan et al. [18] and Jha [19] claim that SWAT is the best among the different hydrological simulation models for simulating the impact of natural and human induced changes in the quantity and quality of surface water. Because SWAT provides flexibility for accommodating enough spatial detail for both biophysical and human influence on the quantity and quality of surface flow, capable of modeling relatively small to very big river basins with high levels of accuracy, SWAT allows creation of a large number of sub-basins to account for topographic variations and to represent heterogenous stresses on the stream networks. Additionally, SWAT’s capability for interfacing with Geographic Information System (GIS) allows for ease in visualization of spatially explicit human infrastructure and in spatial linkage among different biophysical and human factors. SWAT is a physically based, watershed-scale, and continuous time-simulation model, operating on daily and sub-daily time steps [20]. It integrates weather, soil, and topographic characteristics while also providing the flexibility to account for spatially specific land management practices, water abstraction, and discharges to simulate surface hydrology and associated biogeochemical processes [20].

For this work, SWAT was used to generate a high-resolution stream-network, i.e. a topologically and hierarchically connected network of stream segments that form the spatial template to examine the hydrological implications of HI on hydrology. SWAT divides watersheds into smaller sub-basins, and each sub-basin is further categorized into one or more unique hydrologic response units (HRU), each with specific flow generation behavior. HRUs are similar in soil types, land use, slopes, and land management practices. SWAT allows user defined criteria for the number of HRUs in a watershed or sub-basin. However, a user must apply a single criterion for defining HRUs across the entire watershed, so that similar HRUs across the sub-basins in the watershed with similar flow generation characteristics can be identified. SWAT calculates the water balance components (evapotranspiration or ET, surface and sub-surface flow, etc.) for each HRU and aggregates it for a sub-basin. Each sub-basin has a river reach with an inlet within the sub-basin and an outlet that opens to the inlet of the next river reach in the hierarchy of river network in the basin. The surface flow enters the river reach of the sub-basin through the inlet and empties into the inlet of the next connected river reach. Therefore, a unique identifier relates each sub-basin to its river segment within the hierarchy of stream networks. SWAT is well recognized as a robust tool for simulating short-term as well as long-term impacts of changes in land cover and climate on water flow, vegetation dynamics, and water quality variables, including sediment and nutrient across multiple scales [21,[22], [23], [24], [25]]. Moreover, the SWAT model provides flexibility for ex-ante evaluation of the impact of different scenarios on surface hydrology and water quality for a scale appropriate to the user [26,27].

HIs that alter the surface hydrology can be broadly grouped into the two groups: 1) landscape modifications or 2) in-stream modifications. Landscape modifications alter ET and surface flow while in-stream modifications directly alter stream flow through diversions, discharges, or temporary storage of water. These changes can be accommodated at the finest scale of resolution in the model, i.e. the sub-basin. For each sub-basin, streamflow at the outlet $Q_{sb}$ due to changes in the water budget, including HIs, can be generally calculated with the following equations [28]:

(1) Qsb=SWout+Hin+drdt+Hout

(2) SWout=P-ET-dsdt

where P is precipitation, ET is evapotranspiration, ds/dt is the change in surface water storage, and dr/dt is the storage and operation of a reservoir. ${\text{SW}}_{out}$ represents the surface water outflows from each sub-basin whereas ${\text{H}}_{\text{out}}$ denotes human-induced water abstractions and ${\text{H}}_{\text{in}}$ refers to return flows (from off-channel uses). ET and dsdt are parameterized and calculated within the model, whereas the other terms are external inputs to the model. In the case of drdt, however, evaporation from the reservoir’s water surface is calculated within the model, yet storage and releases from the reservoir are determined by reservoir operations schedules, which are model inputs. ${\text{H}}_{\text{in}}$ and ${\text{H}}_{\text{out}}$ are determined by societal demand for water use for different purposes, and we describe these factors in later sections.

Many users of SWAT select to use Arc-SWAT, an ArcGIS interface for SWAT [29], as it provides a convenient interface for visualization of inputs and outputs, drainage networks, and other spatial data. General data needs for the initial set-up are reported in Table 1. Topography was represented by a 30-m resolution digital elevation model (DEM) from USGS National Elevation Dataset [30] to delineate the TNB and ACF basins. A high-resolution (1:24000) dataset of stream reaches from the National Hydrology Database was used to delineate sub-watersheds in each of the basins and each sub-watershed in SWAT, which is associated with a unique stream segment identifier. HRUs for both basins were generated using the National Land Cover Dataset (NLCD [31];) and STATSGO soil map [57], medium resolution (1:250,000 scale) for these basins. Climate inputs were obtained from DAYMET [32] for the study period (1980–2010) for defining the baseline (Table 1).

**Table 1:** Example data inputs and sources for initial SWAT Setup.

Data Input	Spatial resolution	Source
DEM	30m	USGS 3D Elevation Program^a^
Climate	1km	DAYMET^b^
STATSGO	Medium	USDA^c^
LANDUSE	30m	USGS^d^
NHD	High	NHDPlus V2^e^
LAI	Field	Wullschleger et al. [33], Scurlock et al. [34], Hutchinson et al. [35] [36] f.

a https://www.usgs.gov/core-science-systems/ngp/3dep/about-3dep-products-serv.

b https://daymet.ornl.gov.

c https://datagateway.nrcs.usda.gov/.

d https://www.mrlc.gov/data.

e http://www.horizon-systems.com/NHDPlus/NHDPlusV2_home.php.

f https://tde.ornl.gov/RELLAI.tx.

### Identify the entry points and spatial granularity required to represent HI

SWAT provides options for accounting for land and stream-based HIs through land-use changes or through modules to incorporate water intakes, reservoir operations, and released water from point sources, and these constitute different “entry points” into the SWAT modeling framework based on Eq. (1). To exploit these options in SWAT, we developed a detailed space-time database of the current water intakes, discharges, return flows, reservoirs, and land-use changes for TNB and ACF to represent baseline HI and future scenarios of HI within SWAT (see subsequent section). However, SWAT only allows a limited number of any of these human-made changes in each sub-basin. Hence, the number of sub-basins must be increased to accommodate the spatial distribution of HIs. Generally, when developing SWAT models for basins analogous in size to the TNB or ACF basins, sub-watershed size is typically coarse, such as HUC-8 or HUC-10 watershed boundaries and rarely at HUC-12 resolutions [37]. To give an indication of how these spatial units translate into spatial granularity, there are 32 HUC-8, 217 HUC-10 and 1073 HUC-12 watersheds in the TNB, while the ACF has 14 HUC-8, 131 HUC-10 and 677 HUC-12 watersheds. While > 1000 sub-basins seem enough to capture HIs, there are approximately 750 dams and almost 1400 intakes, discharges, and power plants in the TNB alone. In contrast, > 1500 sub-basins appear adequate for accounting around 70 dams and 1900 intakes and discharge for multiple uses. After multiple iterations of sub-watershed granularity, increasing number of sub-watersheds, for TNB and ACF in SWAT, it was evident that a very high-resolution SWAT modeling is warranted for an appropriate accommodation of HI. As a first step, buffers of the spatial database of HI is created, which is then intersected with the sub-watershed created in the SWAT model to make sure that the SWAT models of TNB and ACF has enough sub-watersheds to account the HIs. We decided that 5050 sub-watersheds for the TNB and 2178 for the ACF is needed to account details of HI.

### Generating a space-time database to represent human intervention

In addition to the general inputs needed for the SWAT (See section 2.1), inputs related to HI require much attention and data compilation. These inputs include water abstraction for human consumption, electricity production, industrial activities and the discharges related to the return and treatment of water from these activities. As water abstraction and return vary over space and time, a matrix of stream reaches and infrastructure intake/discharge over different reaches and across various scenarios of HI is generated (Table 2), which describes the baseline land use and reach ID-specific details of daily water supply together with daily water intake (WI) for different energy production portfolios (BE) and their daily return flow (WR) back to the stream. General datasets required to adequately capture the spatial and temporal resolution of HI are provided in Table 3. Of course, the data required to simulate future conditions will also need to approximate the spatial and temporal scales and granularity of datasets used in the baseline calibration process (see Section 2.4).

**Table 2:** Example of the structure of space-time data inputs for representing human infrastructure influences on a river network.

Reach ID	Baseline Land use											
	Water Supply +											
	WI (m^3^s^−1^)						WR(m^3^s^−1^)					
	BE-1	BE-2	BE-3	BE-4	BE-5	BE-6	BE-1	BE-2	BE-3	BE-4	BE-5	BE-6
1												
.												
.												
N

**Table 3:** Examples of data and sources of information used to develop a space-time database to represent human infrastructures in hydrologic models.

Human Infrastructure Dataset	Description	Examples of sources
Dams and reservoirs	Spatial coverage of dams and reservoirs. Reservoir operation determined using schedules or through calibration process (also see Section 2.4)	National Anthropogenic Barrier Dataset^a^; NHDPlus V2 waterbodies^b^; McManamay et al. [38]
Water abstraction	Locations and reported water usage for public water supply intakes, irrigation siphons, diversions, industrial facilities, and groundwater wells (if the hydrologic model accommodates groundwater usage). Water usage may be reported at decadal, annual or monthly timesteps. Capacity may be reported instead of usage. Note that data may be sensitive.	State Water Permitting Department Websites: - Tennessee Department of Environment and Conservation^c^ - North Carolina Center for Geographic Information and Analysis^d^ - Mississippi Department of Environmental Quality^e^ - Georgia Environmental Protection Branch^f^ - North Georgia Metropolitan Planning District^g^ - Florida Department of Environmental Protection^h^
Point discharges	Locations and reported discharges of water treatment facilities or industrial point-source discharges	Facility Registration System, NPDES^i^ and same sources provided for water abstraction
Power plants	Locations of electricity generating stations according to technology types. Water usage estimates vary monthly for each location or for each fuel type, primary mover, and cooling technology.	Energy Information Administration, Forms 860^j^and 923^k^.

a https://www.sciencebase.gov/catalog/item/56a7f9dce4b0b28f1184dabd.

b http://www.horizon-systems.com/NHDPlus/NHDPlusV2_home.php.

c http://www.tn.gov/environment/dataviewers.shtml.

d https://it.nc.gov/center-geographic-information-and-analysis-cgia?tabid=55.

e http://opcgis.deq.state.ms.us/MSWRDataCompendium/.

f http://epd.georgia.gov/watershed-protection-branch.

g http://www.northgeorgiawater.org/plans/water-supply-and-water-conservation-management-plan.

h https://ca.dep.state.fl.us/mapdirect/.

i http://www.epa.gov/enviro/html/fii/index.html.

j https://www.eia.gov/electricity/data/eia860/.

k https://www.eia.gov/electricity/data/eia923/.

We utilized available point source units in TNB and ACF to define either water abstraction from the stream or point source discharge (including return flows of the water taken out of the stream), where return flows are expressed as positive values and water abstraction is expressed as negative values. SWAT only allows one-point source per sub-watershed; therefore, if the location of a point source was close to water abstraction, only one location was used, and water use was expressed as net-intake/net-discharge. If the water intake and point source discharge locations were not in proximity, water intakes were represented as water storage ponds. In certain cases, however, SWAT cannot accommodate multiple confounding infrastructures, such as where a sub-basin contained a water intake, point source discharge, and a dam. In these cases, water abstraction was shifted to the inlet of the immediate downstream segment (sub-watershed). The spatial locations of all water infrastructures were determined from numerous sources and spatially joined to specific stream segments and corresponding sub-basins. Estimates of water extraction, return flows, and discharges were obtained from state-water permitting agencies, the Environmental Protection Agency, or energy producer databases (Table 3). Generally, the temporal resolution of reported values varied by state and source, and we attempted to use the finest temporal resolution available (e.g., monthly averages). In some cases, water use or discharge had to be estimated based on technologies (e.g., power plants, see Averyt et al. [39]) or adjusted based on reported capacities of infrastructures (e.g., water intakes). In general, reasonably accurate information on daily operations of dams or reservoirs were difficult to obtain for multiple reasons. However, an accurate representation of reservoir operations is critical for the hydrologic simulation of the basin as well as stream scale hydrology. The reservoir option available in SWAT was used for creating reservoirs and dams in the study area, and reservoir operation information was derived using a combination of iterative SWAT simulation and reservoir storage/release described in Section 2.4.

#### Databases representing future human infrastructures

Future changes in land use, electricity and water demands, and infrastructures can be represented using scenarios. As stated earlier, the spatial and temporal resolution of these data need to approximate that used to calibrate the hydrologic model. An example of a scenario-based approach used to generate spatially explicit future scenario data is provided by McManamay et al. [40]. The approach downscales national-level Shared Socioeconomic Pathways for the region [41,58] to derive city-scale scenarios of population growth, electricity and water demand, future energy portfolios, and future water infrastructure expansion. For hydrologic simulation, we developed a total of 40 scenarios in accordance with the scenarios outlined in McManamay et al. [40,42] with two specific timeframes, 2030 and 2050. Generally, the scenarios consisted of projected population, city expansion and city land use, two climatic regimes based on extreme value theory (extreme wet and extreme dry), 5 potential energy supply mix futures, and 2 potential water supply futures.

While spatially explicit infrastructure scenarios are described in McManamay et al. [40], we briefly provide methods to generate extreme wet and dry projected climate scenarios in this paper. We decided to estimate extreme future wet and dry scenarios using extreme value theory based on historical climatological records for a couple of reasons. First, the lack of downscaled future climate extremes at the appropriate resolution, a requirement for our study, prevented the use of downscaled climate scenarios in the study. Second, climate extremes are more suitable to understanding the resilience of infrastructure, but may not be adequately captured in downscaled climate scenarios emerging from Representative Concentration Pathways (RCP) (e.g., Hurricane Sandy and Hurricane Harvey). We estimated extreme value distribution for the TNB and ACF basins using data from Daymet database (1980–2010). A threshold value of 1 mm of precipitation amount was defined as a wet day, whereas a dry spell was defined as the number of consecutive days with less than 1 mm precipitation. First, for the sub-basins in TNB and ACF, precipitation extreme was chosen using Block Maxima and two new produced data sets were fitted on both the Generalized Extreme Value Distribution with a return period of 100 years [43,54].

### Calibrate and validate the model

**Fig. 2:**
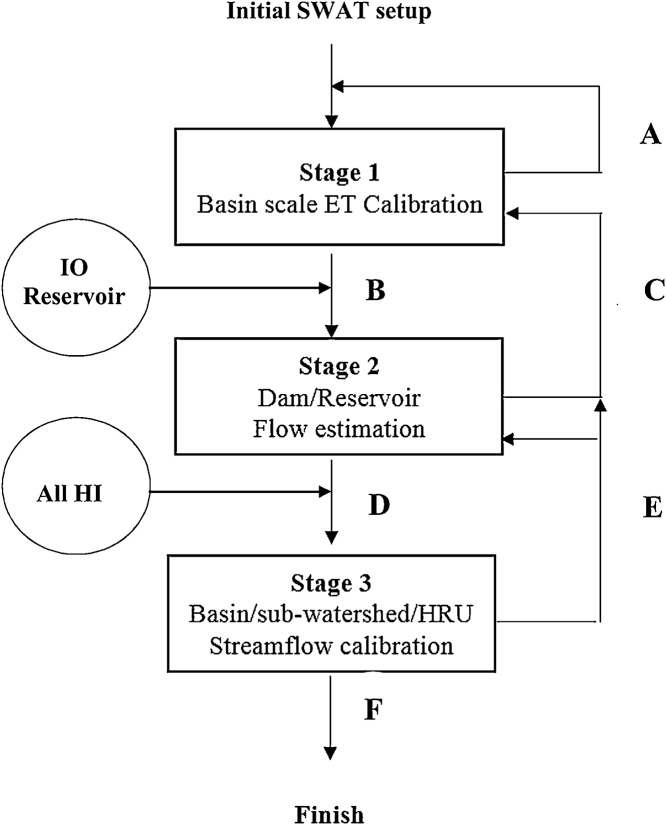
Sequential and circular three-stage calibration. The following are thresholds used in the decision-tree calibration approach: **IO** – inflow (I) to the reservoir and outflow (O) from the reservoir; **A**= ET ≤ 70% of reported ET. **B**= ET >70 % of reported ET. **C** = Reservoir outflow ET ≤ 70% estimated outflow. **D** = Reservoir outflow ET > 70% estimated outflow. **E** = Streamflow ≤ 70% USGS gage flow. **F** = Streamflow close to USGS gage flow Nash-Sutcliffe coefficient of efficiency is ≥ 0.9.

Calibration followed a sequential and circular three-stage approach (Fig. 2), where multiple iterations were attempted to improve model parameters (ET and ds/dt) while also incorporating HI structure. In each stage, thresholds for model performance are used in a decision tree to determine progression towards subsequent steps (Fig. 2). In Stage 1, ET for the basin was calibrated against reported values from literature for the region. This ensures that any simulations to downstream infrastructures (e.g., reservoirs) are reasonable. In Stage 2, daily water inflows and releases from the reservoirs were estimated to develop reservoir operation modules and estimate downstream discharge releases from dams. We then updated the model with these discharges and moved to Stage 3, where parameters specific to sub-watersheds and HRUs are calibrated to minimize differences between simulated values and empirical observations at stream gaging stations (e.g., US Geological Survey stream gages).

#### Stage 1: calibrate ET for the basin

Calibration of evapotranspiration (ET) was conducted in multiple steps, initially at the basin-scale to identify gross basin-level water budget components not influenced by major infrastructures or reservoir operations. At this stage, the aim of calibration was to simulate ET as close as possible to the reported values in the literature for the study area. Earlier studies showed that radiation-based method for potential ET is suitable for the study area, so we selected Priestley-Taylor equations to calculate potential ET [59]. We observed that our simulated ET value was less than reported values, specifically 64% of values reported in TNB and 67% of values for the ACF; however, we also observed a close relationship between ET and leaf area index (LAI) both for the TNB and AFC basins and for randomly selected sub-basins in TNB and ACF. Therefore, LAI was increased by 10% for shifting the ET curve upward and aligning our simulated ET values with previously reported estimates. The LAI values, specific to TNB, were the mean value from Wullschleger et al. [33], Scurlock et al. [34], and Hutchinson et al. [35] along with the daily progression of LAI over the growing season for the years 1992–2000 from Hanson et al. [36] and Hanson et al. [55] (data can be found at https://tde.ornl.gov/RELLAI.txt). As the landscape in TNB and ACF is dominated by similar forest types, and are under similar climatology, the same LAI values were applied for ACF.

#### Stage II: estimate reservoir operations

Reservoir operation algorithms have been widely developed for hydrologic models ranging from individual dams to basin-levels, and ultimately global-scale applications [[44], [45], [46], [47], [48], [49], [50]]. Accurate simulation of hydrology in most human-dominated systems requires incorporation of reservoir operation algorithms; however, developing accurate algorithms is an intensive process and difficult for highly regulated systems with many water control structures. Furthermore, one must consider the objective of developing reservoir operations, whether aimed at optimizing future reservoir operations or accurate hydrologic simulation of human-dominated systems. In our case, we aimed to simulate hydrology accurately in a model that incorporates highly detailed information on HI, in order to examine the future effects of HI on local-to-regional water availability.

(3) Flow (m3s-1) at G2 = Flow from the Damdrdt(m3s-1) + Flow from the sub-basin C(m3s-1)

**Fig. 3:**
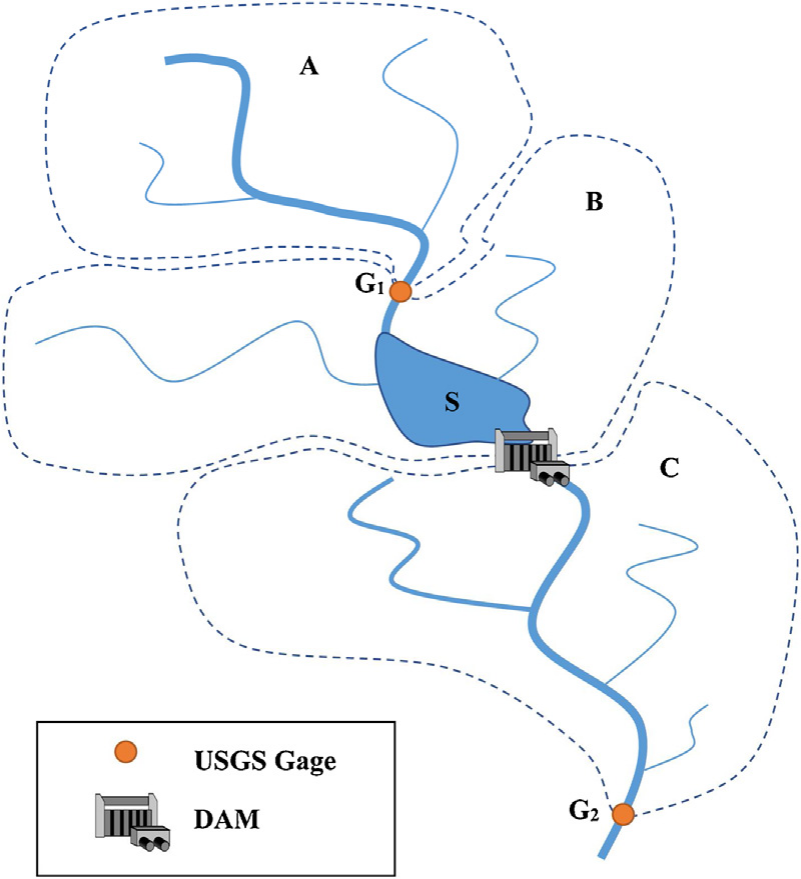
Estimating reservoir outflow from SWAT output. **A** and **B**- upstream sub-basins contributes water to the reservoir with average storage **S**. **G1 -**Upstream gage measures the flow from sub-basin A. **C** – A downstream sub-basin contribute to USGS gage (**G2**).

Therefore, we developed a simplified process for incorporating reservoir operations in our hydrologic modelling platform (Fig. 3). Essentially, the dr/dt parameter in Eq. (1) is derived by calculating the differences between simulated inflows to each dam and the observed outflows from the dam at any desired timestep (daily, monthly) using Eq. (3). This procedure requires simulation and calculation of reservoir releases at the most upstream dam in the basin first, followed by iterative simulation and calculation of outflows for the nearest downstream dam, and so on. The first step requires identifying the most proximate US Geological Survey (USGS) stream gaging stations occurring upstream and downstream of a reservoir. Second, the sub-watersheds contributing to the dam and contributing to the gaging stations are delineated for estimating inflows. The SWAT model calibrated in Stage 1 is then used to simulate inflows to the dam and inflows for drainage areas contributing to areas between the dam and the nearest downstream USGS gage. After accounting for inflows due to drainage area differences, the net difference in flow between the simulated inflow to the dam and the observed outflows from the dam equal the dr/dt. Reservoir outflow for the historical precipitation extremes were used for representing reservoir flow for future precipitation extremes.

#### Stage III: Basin/Sub-watershed streamflow calibration

After reservoir operations have been estimated and HI inflows and outflows have been accommodated, soil and catchment hydrologic parameters require adjustment for appropriate calibration. For example, the soil moisture augmentation parameter is adjusted to increase soil water availability in order to compensate for lower ET demand relative to reported ET values. Thus, initial condition curve numbers (CN2) were decreased by 10% for all HRUs in the basin, which essentially increases water movement into the soil profile. The soil available water capacity (SOL_AWC) was increased by 20% to augment the water storage in the soil profile, permitting the water entering the soil to be available for crop use. Furthermore, the base flow parameter, ALPHA_BF, was reduced from 0.048 to 0.02 based on an analysis of USGS stream gage data and an application of a base flow separation program. Subsequently, the soil evaporation compensation factor (ESCO) and plant uptake compensation factor (EPCO) parameters were adjusted to better represent ET demands. To address event timing, the parameter that controls daily runoff as a fraction of total available water, surface runoff lag coefficient (SURLAG), was changed to 1.

**Fig. 4:**
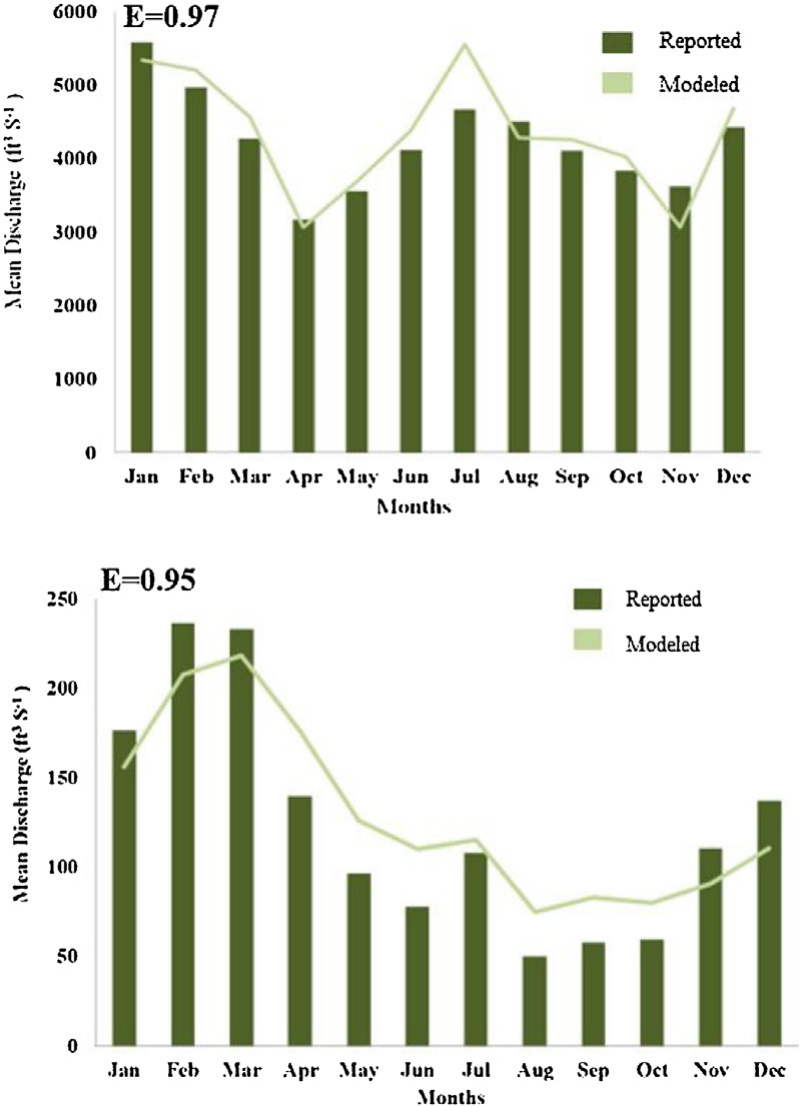
A. Monthly mean discharge at USGS gage # 03518300 in TNB over.1985–2000 B. Monthly mean discharge at USGS gage # 02344700 in ACF over 1985–2000.

**Fig. 5:**
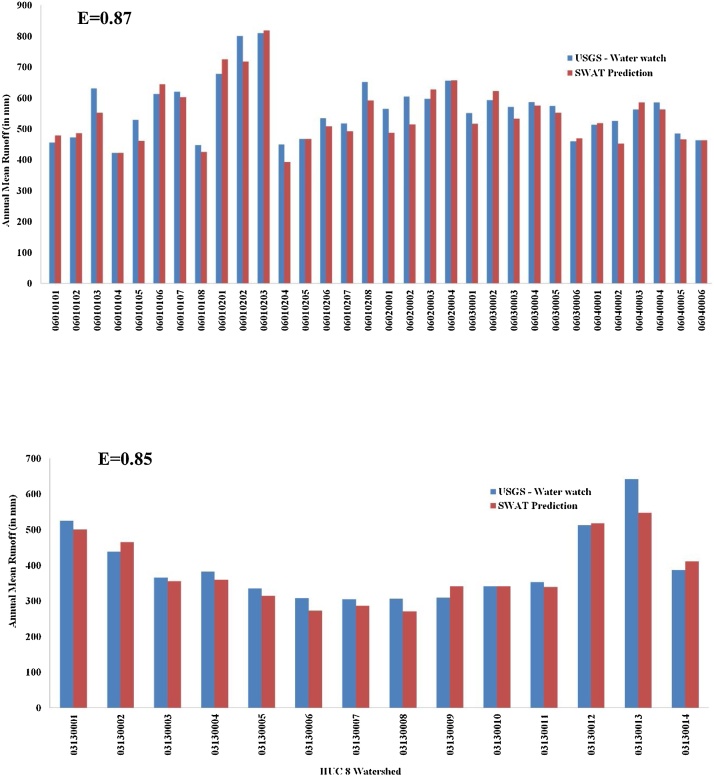
A. Validation result of annual mean runoff for HUC-8 watersheds in TNB and USGS water watch data. B. Validation result of annual mean runoff for HUC-8 in ACF and USGS water watch data.

We calibrated and validated the SWAT generated streamflow against the reported streamflow values of the USGS gages within the TNB (USGS GAGE number 03518500) and ACF basins (USGS GAGE number 02344700) (Fig. 4). The baseline simulation period, 1980–2010, was divided into three groups, 1) five years applied for model warm-up runs, and from the remaining 26 years, 2) first 16 years (1985–2000) for calibration and 3) the remaining 10 years (2001–2010) used for validation of the model. Additionally, USGS water-watch data (https://waterwatch.usgs.gov/) was used to evaluate the average annual flow at HUC-8 watershed outlet for the validation period (Fig. 5). Model performance was evaluated using a commonly used error measure in modeling, the Nash-Sutcliffe coefficient of efficiency (E) [51], which was calculated as follows:

(4) $$\text{E}=1-\sum_{i=1}^{n}\frac{{\left(X^{O}-X^{S}\right)}^{2}}{{\left(X^{O}-X^{Mean}\right)}^{2}}$$

where $X^{O}$ and $X^{S}$ is individual observed and simulated values, respectively, and $X^{Mean}$ is the mean of observed values.

As the calibrated and validated SWAT model for TNB and ACF will be applied to understand the changes in monthly/seasonal/yearly impact of flow values by human modification of surface hydrology, the efficiency measure for TNB and ACF model simulations were calculated for monthly values. Results of validation of the ACF and TR basin SWAT model are given in Fig. 4, Fig. 5.

### Model simulations

**Fig. 6:**
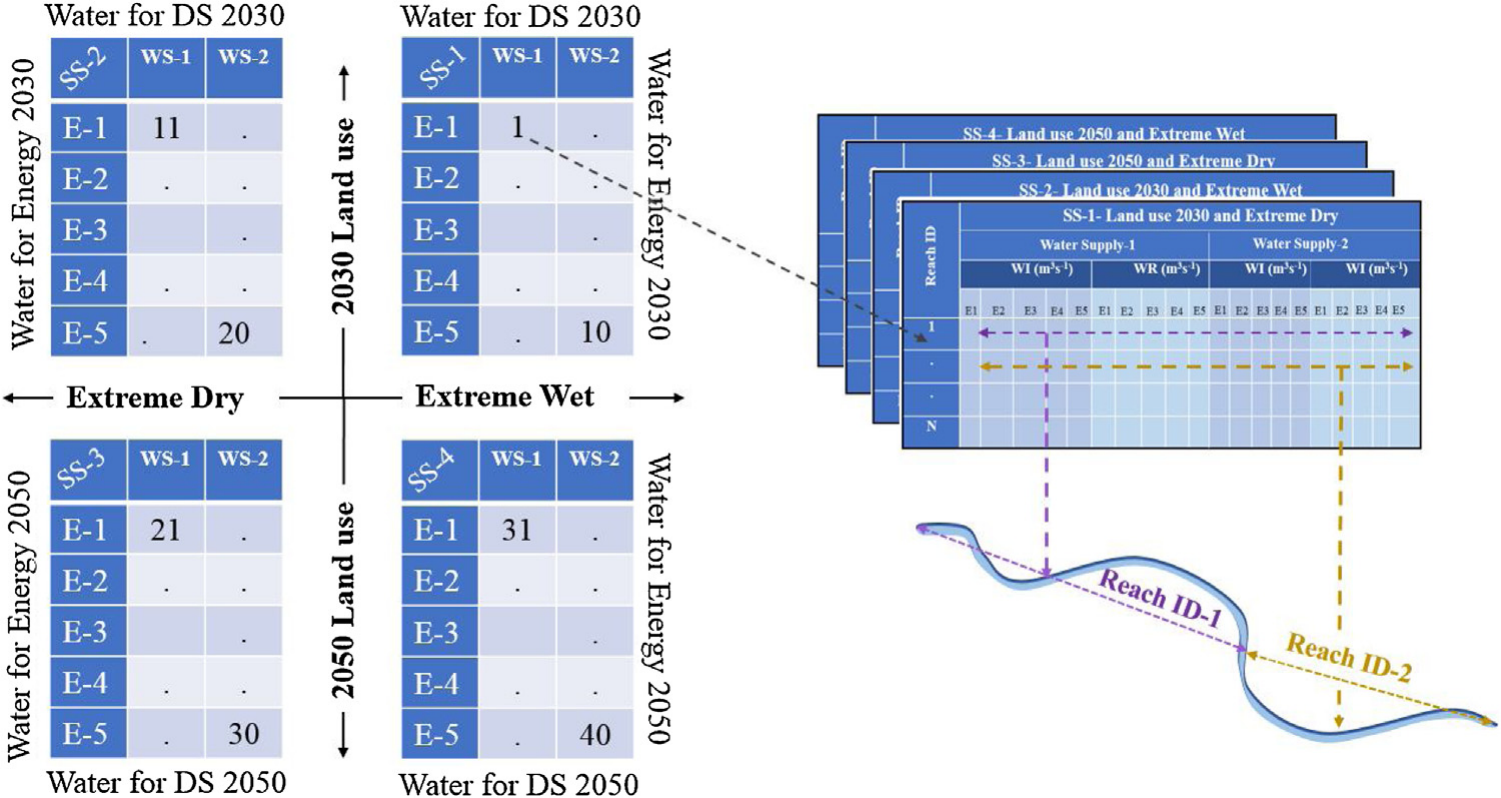
Implementing spatially explicit and temporally specific HI scenarios in SWAT. SS: Scenario Set; DS: Domestic supply (WS-1 and WS-2); WI: Water Intake; WR: Return flow; E1-E5: water need for five energy portfolios.

After calibration and validation of the TNB and ACF models, models were applied for simulating the impact of future water and energy infrastructures and urban expansion on local and regional surface hydrology in TNB and ACF basins. The future development of water and energy infrastructure and growth of urban areas in terms of water intake and return flows were expressed using 40 scenarios for two future time periods, 2030 and 2050, and under two extreme water availability settings, extreme wet and extreme dry. The 40 future scenarios were grouped into four sets of 10 scenarios for smooth implementation in SWAT model for TNB and ACF (Fig. 6). As land use is one of the variables that define HRU in SWAT; and weather variables, in the extreme wet and dry condition, are also principal inputs to the SWAT; four copies of the validated TNB and ACF models were created to simulate each of the scenarios corresponds to the four-quadrants in Fig. 6. Accordingly, land use and weather inputs in the model are updated corresponding to each of the quadrants. Additionally, a table that represents amount of water intake and returns flow for each of the reaches in TNB and ACF models of SWAT at a daily time step for each of the ten scenarios in a set is created (Fig. 6). Once is the time step is completed, water intake and return flow in each scenario in a set are applied to corresponding reaches in the TNB and ACF models at a scenario-specified frame of time and run in the simulation. Similarly, all forty scenarios are simulated for TNB and ACF.

## Summary

Across the globe, unsustainable management of available water has created a new normal of demand-supply mismatch, i.e., human-induced water stress. Our ability to correctly estimate these mismatches in space (hot spots of water stress) and time (hot moments of water stress) is limited by the tools at our disposal. Accurate and easily accessible and usable information for water managers is essential for sustainable management of regional water resources. Hydrology models are part of this information source. It is essential, however, that hydrology model-assisted decision-making for sustainable regional water resource management account for human infrastructures, their interactions, and feedbacks in the modeling exercise. Here, we demonstrated an approach to show the usefulness of already available hydrology models to account HI in regional hydrology modeling. The SWAT model was used as an example model to illustrate a generalizable hydrologic modeling framework that exploits the flexibility in the current hydrologic model to account both natural drivers and HI. We applied the proposed approach to two basins with different stages of urbanization in the southeastern US, the Tennessee River basin, and the Apalachicola-Chattahoochee-Flint River basin. A baseline HI database for the study region was developed to account the current human influences on surface hydrology, including water abstraction, storage, diversion, and return flow. A scenario-based spatially-explicit future demand estimated for water needs for varied uses by the society was used to account the future influences of HI. It was clear that a higher resolution modeling of the basin that goes beyond the traditional watershed boundaries is vital to accommodate the local as well as the regional impact of HI on surface hydrology. The calibration and validation showed model performance at an acceptable accuracy level. However, by including HI in hydrology models, another layer of uncertainty, uncertainty in coupled socio-hydrological models, adds to existing biophysical model uncertainty.

## Disclaimer

This manuscript has been authored by UT-Battelle, LLC under Contract No. DE-AC05-00OR22725 with the U.S. Department of Energy. The United States Government retains and the publisher, by accepting the article for publication, acknowledges that the United States Government retains a non-exclusive, paid-up, irrevocable, world-wide license to publish or reproduce the published form of this manuscript, or allow others to do so, for United States Government purposes. The Department of Energy will provide public access to these results of federally sponsored research in accordance with the DOE Public Access Plan (http://energy.gov/downloads/doe-public-access-plan).

## Supplementary material and/or additional information

[OPTIONAL]. We also give you the option to submit both supplementary material and additional information. Supplementary material relates directly to the work that you have submitted and can include extensive excel tables, raw data etc. We would also encourage you to include failed methods or describe adjustments to your methods that did not work. Additional information can include anything else that is not directly related to your method, e.g. more general background information, useful links etc. Introduction is not a section included in the MethodsX format. This information could be moved to the end under Additional Information.
